# Cellular Mechanisms of Melatonin: Insight from Neurodegenerative Diseases

**DOI:** 10.3390/biom10081158

**Published:** 2020-08-07

**Authors:** Dongmei Chen, Tao Zhang, Tae Ho Lee

**Affiliations:** Fujian Key Laboratory for Translational Research in Cancer and Neurodegenerative Diseases, Institute for Translational Medicine, School of Basic Medical Sciences, Fujian Medical University, Fuzhou 350122, Fujian, China; taozh@fjmu.edu.cn

**Keywords:** melatonin, neurodegenerative disease, Alzheimer’s disease, Parkinson’s disease, Huntington’s disease

## Abstract

Neurodegenerative diseases are the second most common cause of death and characterized by progressive impairments in movement or mental functioning in the central or peripheral nervous system. The prevention of neurodegenerative disorders has become an emerging public health challenge for our society. Melatonin, a pineal hormone, has various physiological functions in the brain, including regulating circadian rhythms, clearing free radicals, inhibiting biomolecular oxidation, and suppressing neuroinflammation. Cumulative evidence indicates that melatonin has a wide range of neuroprotective roles by regulating pathophysiological mechanisms and signaling pathways. Moreover, melatonin levels are decreased in patients with neurodegenerative diseases. In this review, we summarize current knowledge on the regulation, molecular mechanisms and biological functions of melatonin in neurodegenerative diseases such as Alzheimer’s disease, Parkinson’s disease, Huntington’s disease, amyotrophic lateral sclerosis, vascular dementia and multiple sclerosis. We also discuss the clinical application of melatonin in neurodegenerative disorders. This information will lead to a better understanding of the regulation of melatonin in the brain and provide therapeutic options for the treatment of various neurodegenerative diseases.

## 1. Introduction

Melatonin (*N*-acetyl-5-methoxytryptamine) was first discovered six decades ago and is a multifunctional hormone mainly produced by the pineal gland in response to darkness [1]. As it is a circadian rhythm-regulated hormone, the secretion of melatonin is tightly regulated. The concentration of melatonin in the night is up to 10 times higher than that in the daytime. Melatonin secretion starts to increase at around 9 p.m., reaching its peak level during the overnight hours, and decreases the next morning [2]. Melatonin production is decreased with aging and in certain diseases, including neurodegenerative diseases, indicating that the deregulation of melatonin may cause the development or progress of human diseases. In addition to its role in sleep and circadian rhythms, melatonin has been shown to exert neuroprotective effects, antioxidant defense, anti-inflammatory effects, and anti-apoptotic activity in both cellular and animal models [3,4]. Extensive evidence has shown that melatonin has preventive and clinical effects on various diseases, including cancer and neurodegenerative diseases, and can even attenuate viruses such as severe acute respiratory syndrome coronavirus 2 (SARS-CoV-2), which causes coronavirus disease 2019 (COVID-19) [5,6,7,8].

Neurodegenerative diseases cause progressive loss of brain functions and eventually result in severe disability and death with aging populations. Many neurodegenerative diseases share overlapping clinical features such as cognitive deficits, motor system defects and sleep disorders, and common molecular mechanisms including signaling pathways, protein aggregation, and protein seeding and spreading from one region to another [9]. Melatonin has been shown to ameliorate the neuropathological features of various neurodegenerative diseases, and melatonin supplementation contributes to alleviating cognitive and/or motor impairments as well as sleep problems in patients. In this review, we discuss the molecular mechanisms by which melatonin protects against neurodegenerative diseases and the clinical application of melatonin in these devastating diseases.

## 2. Biosynthesis of Melatonin

**Figure 1:**
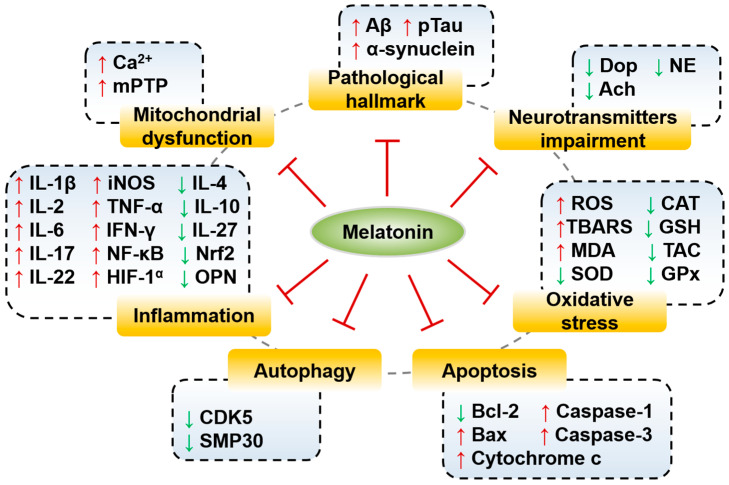
Schematic representation of melatonin therapy by targeting molecular signaling pathways in neurodegenerative diseases with different pathogenic mechanisms. Melatonin inhibits pathological hallmarks, oxidative stress, inflammation, neurotransmitter impairment, mitochondrial dysfunction, apoptosis and autophagy. mPTP, mitochondrial permeability transition pore; Dop, dopamine; Ach, acetylcholine; NE, norepinephrine; TBARS, thiobarbituric acid reactive substances; CAT, catalase; GSH, glutathione; TAC, total antioxidant capacity; GPx, glutathione peroxidase; MDA, malondialdehyde; ↑, induction; ↓, reduction.

Melatonin is mainly produced from pinealocytes in the pineal grand and also synthesized in other organs such as the bone marrow, retina, skin and gastrointestinal tract (GIT). The whole procedure of melatonin biosynthesis occurs principally in four steps including hydroxylation, decarboxylation, acetylation and methylation (Figure 1) [2,10]. Firstly, L-tryptophan is turned into 5-hydroxytryptophan by tryptophan hydroxylase with the stimulus of dark. Secondly, 5-hydroxytryptophan is decarboxylated into 5-hydroxytryptamine (serotonin) via the presence of 5-hydroxytryptophan decarboxylase. Vitamin B6 is required during the step of decarboxylation. The next step is the acetylation of the serotonin by serotonin *N*-acetyl transferase (the rate-limiting enzyme), which converts it into *N*-acetylserotonin. Protein kinase A (PKA) plays a critical role in the rate-limiting step by activating serotonin *N*-acetyl transferase [11]. Finally, *N*-acetylserotonin is methylated to melatonin through hydroxyindole *O*-methyl transferase with folate. Melatonin stimulates the suprachiasmatic nucleus (SCN), resulting in norepinephrine secretion. Norepinephrine acts on the endoplasmic reticulum of pinealocytes and activates α-1/β adrenoceptors, leading to an increased concentration of calcium (Ca^2+^) in the cytosol. Furthermore, the membrane-bound adenylyl cyclase is activated to produce intracellular cAMP, which eventually binds to PKA and increases PKA activity [12].

## 3. Function of Melatonin

Melatonin has a variety of functions, including circadian rhythm regulation, antioxidant activity, and anti-inflammation and anti-apoptotic effects. Melatonin plays a critical role in the circadian rhythm in most mammals depending on light/dark cycles [2]. Light inhibits melatonin synthesis and secretion, while the absence of light stimulates melatonin production and secretion. It has been reported that melatonin exerts effects on attenuating circadian disruption through the regulation of clock genes in vivo [2,13]. Melatonin increases the expression levels of cryptochrome 2 (CRY2), period circadian protein homologue 1 (PER1) and brain muscle ARNT-like 1 (BMAL1), which are associated with neurodegenerative diseases. Since melatonin-mediated clock gene expression shows opposite results under certain conditions, the exact mechanisms have not been elucidated yet [4]. Melatonin acts as a free radical scavenger and has antioxidant functions. It has been shown that melatonin has a protective effect on cancer, epilepsy and neurodegenerative disorders by blocking oxidative stress in vivo and in vitro [14,15,16]. Melatonin has been found to increase the expression and activity of enzymes, such as glutathione peroxidase (GPx), superoxide dismutase (SOD) and catalase (CAT), involved in antioxidant function [17]. Moreover, melatonin reacts with reactive oxygen species (ROS) and reactive nitrogen species (RNS), resulting in it being converted to an antioxidant, *N*1-acetyl-*N*2-formyl-5-methoxykynuramine (AFMK), through oxidization [18]. Melatonin also has an important role in anti-inflammatory effects. Melatonin has been documented to attenuate pathogenic inflammation by regulating various pathways, such as decreasing the secretion of cytokines (interleukin-1 (IL-2), interferon-gamma (IFN-γ) and tumor necrosis factor-alpha (TNF-α)) and increasing the amounts of cytokines (IL-4, IL-10 and IL-27). It has been shown that melatonin alleviates the secretion of proinflammatory cytokines by inhibiting nuclear factor kappa B (NF-κB) [19]. Moreover, melatonin inhibits the expression of cyclooxygenase-2 (COX-2), which is a proinflammatory factor, in neurodegenerative diseases [20]. In addition, melatonin suppresses apoptosis by modulating Bcl2/Bax and reducing the expression and activity of caspase-3, suggesting that melatonin regulates apoptotic function in the protection against cancer and neurodegenerative diseases [21,22,23].

## 4. Action Mechanism of Melatonin

Melatonin can exert its downstream effects via binding to specific receptors or direct association with its substrates. Melatonin acts through membrane receptors, which are mainly located in the central nervous system, including melatonin receptor type 1a (MT1), melatonin receptor type 1b (MT2) and melatonin receptor type 1c (MT3) [24,25]. The activation of MT1 and MT2, which are members of the G-protein coupled receptors, leads to the physiological or pathophysiological effects of melatonin in sleep disorders, pain, anxiety, depression and neurodegenerative diseases [26,27]. MT3 has been purified and characterized as a quinone reductase 2 enzyme; however, little is known about the effects of melatonin mediated by MT3 [28]. Melatonin can enter the nucleus and interact with the transcription factor retinoid-related orphan receptor-alpha (RZR/ROR). Its interaction plays a crucial role in immune modulation and antioxidant enzyme regulation through RORα [29,30]. Moreover, melatonin binds to intracellular proteins such as tubulin, calmodulin (CaM) and calreticulin [31,32,33]. For example, melatonin directly binds to CaM with high affinity and antagonizes the binding of Ca^2+^, which is an intracellular secondary messenger, to CaM [24,34]. These interactions are related to the regulation of enzymes such as cAMP phosphodiesterase, CaM-kinase II and nitric oxide synthase [35,36].

## 5. Effects and Molecular Mechanisms of Melatonin in Neurodegenerative Diseases

Cumulative evidence has suggested that melatonin has preventive and therapeutic effects on many neurodegenerative disorders [6,37]. Neurodegenerative diseases have been reported to share common pathophysiological features, such as disruption of the circadian rhythm, increased oxidative stress, neuroinflammation, neuronal loss, autophagic deficiency and mitochondrial dysfunction [10,38]. The level of melatonin decreases in elderly individuals, which may contribute to the development of neurodegenerative disease [39,40]. Moreover, increasing evidence has shown that melatonin has neuroprotective roles in neurodegenerative disorders with few side effects, even at high doses (Figure 1) [41,42]. In this part, we summarize the information of each neurodegenerative disease including the effects of melatonin, cell/animal model, signaling pathway affected, concentrations (physiological and pharmacological) and references (Table 1, Table 2, Table 3, Table 4, Table 5 and Table 6).

### 5.1. Melatonin and Alzheimer’s Disease

Alzheimer’s disease (AD) is characterized by progressive cognitive impairment and other neurobehavioral deficits and is widely known as the most common neurodegenerative disease in the elderly population [43,44]. The pathological features of AD have been identified as extracellular senile plaques (SPs), which mainly consist of accumulated β-amyloid (Aβ), and intracellular neurofibrillary tangles (NFTs), which mainly consist of aggregated, abnormally hyperphosphorylated tau [45,46,47]. Melatonin levels in the serum and cerebrospinal fluid (CSF) are lower in AD patients than those in age-matched control subjects [48,49,50]. Moreover, melatonin supplementation was shown to alleviate the dysregulation of the circadian rhythm and improve cognition in AD patients [51,52].

**Figure 2:**
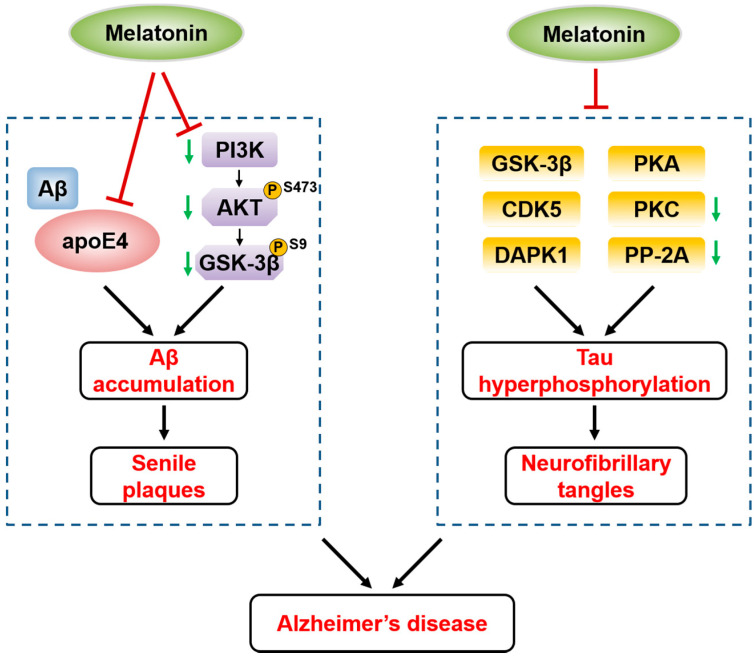
The regulation of Aβ and tau through different kinases in Alzheimer’s disease (AD), which is attenuated by melatonin. Melatonin directly binds to and inhibits apoE4, which enhances Aβ toxicity via combination with Aβ, thereby reducing Aβ pathology in AD. Moreover, melatonin suppresses the reduction of PI3K activity, pSer473 on Akt and pSer9 on GSK-3β, leading to the reduction of Aβ aggregation in AD. Melatonin attenuates tau pathology by regulating several kinases such as GSK-3β, CDK5, PKC, PKA and DAPK1, and the protein phosphatase PP-2A in AD. ↓, reduction.

Aβ peptides are composed of 39–43 amino acid residues and derived from amyloid precursor protein (APP). APP is cleaved by nonamyloidogenic or amyloidogenic processing. These processes are mediated by different secretases [53]. Aβ is generated via amyloidogenic processing by β- and γ-secretase. Melatonin has been reported to inhibit Aβ production and aggregation both in vivo and in vitro [54,55,56,57]. Melatonin reduces the aggregation of Aβ via direct interaction with Aβ (1–40) and Aβ (1–42) and protects neurons against Aβ toxicity [58]. In addition, combinations of Aβ and apolipoprotein E4 (apoE4) synergistically aggravate Aβ neurotoxicity, which can be prevented by melatonin through interactions with apoE4 [59]. In Aβ-induced animal models, melatonin reduces Aβ production and inhibits apoptosis by decreasing caspase-3 activity and elevating B cell lymphoma-2 (Bcl-2) expression in the brain [21,60]. Moreover, melatonin not only changes the levels of caspase-3 and Bcl-2 but also decreases the expression or activity of GSK-3β and increases the protein phosphatase-2A (PP-2A) level [61]. Furthermore, melatonin may reduce Aβ accumulation via GSK-3β inhibition mediated by upregulating the PI3K/Akt signaling pathway, which is inactivated by Aβ (1–42) treatment [62]. This finding is consistent with the evidence that the activation of GSK-3β leads to synaptic and memory impairments, whereas GSK-3β inactivation improves synaptic and memory dysfunctions [63,64]. In addition, it has been reported that PI3K increases the phosphorylation of Akt at the Ser473 site, which leads to the increased phosphorylation of GSK-3β at Ser9, thereby inactivating GSK-3β [65]. Therefore, these results suggested that melatonin increases PI3K activity, Akt phosphorylation on Ser473 and GSK-3β phosphorylation on Ser9, thereby reducing Aβ aggregation, rescuing synaptic dysfunction and attenuating memory deficits in AD (Figure 2).

Tau is a microtubule-associated protein that is involved in stabilizing the microtubule cytoskeletal network and promoting microtubule assembly [43,66]. The hyperphosphorylation of tau disrupts the binding of tau to microtubules, thereby disrupting the stability of microtubules [43,66]. More than 30 phosphorylation sites of tau have been identified in AD brains. Hyperphosphorylated tau aggregates into paired helical filaments (PHFs) with abnormal conformations and eventually forms NFTs in AD [43,66]. Melatonin has been found to significantly ameliorate tau hyperphosphorylation induced by wortmannin, calyculin A (CA) and okadaic acid in different neuronal cell lines [67,68]. Melatonin also efficiently reduces tau hyperphosphorylation induced by Aβ (1–42), kainic acid, wortmannin, CA, isoproterenol and constant illumination in animal models [62,68,69,70,71,72].

Multiple protein kinases have been shown to phosphorylate tau and drive tau aggregation in neurofibrillary tangles in AD, and protein phosphatases are also involved in the regulation of tau phosphorylation [73]. Melatonin attenuates tau hyperphosphorylation by regulating proline-directed serine/threonine kinases such as GSK-3β and cyclin-dependent kinase 5 (CDK5); non-proline-directed serine/threonine kinases, including PKC, PKA and death-associated protein kinase 1 (DAPK1); and protein phosphatase PP-2A (Figure 2) [61,71,72,74,75,76,77,78]. Melatonin has been shown to effectively attenuate tau hyperphosphorylation and oxidative stress by inactivating GSK-3β in cell and animal models of AD [61,72,74]. Melatonin inhibits the activity of CDK5, thereby reducing tau hyperphosphorylation in a rat model [75]. Furthermore, melatonin decreases CDK5 expression and the cleavage of p35 to form p25, which leads to the formation of a stable active CDK5/p25 complex [79]. In addition, it has been discovered that melatonin decreases tau hyperphosphorylation, inhibits oxidative stress and finally attenuates memory deficits via increasing the activity of PP-2A in a rat model [78]. Recently, melatonin was found to directly bind to DAPK1 and promote DAPK1 protein degradation through the ubiquitin-mediated proteasome pathway, resulting in increased Pin1 activity and eventually decreased tau hyperphosphorylation and tau-related pathologies [80]. Pin1 is a phosphorylation-specific peptidyl prolyl cis/trans isomerase and has protective effects on tau-related pathology, suggesting that the melatonin–DAPK1–Pin1 axis regulates AD [81,82,83,84].

### 5.2. Melatonin and Parkinson’s Disease

Parkinson’s disease (PD) is a chronic and neurodegenerative disease with motor symptoms such as resting tremor, bradykinesia, rigidity and postural imbalance and nonmotor symptoms such as constipation, dysosmia, sleep problems and cognitive impairment [85,86]. The pathological hallmarks of PD have been defined as dopamine (Dop) depletion resulting from the progressive loss of nigrostriatal dopaminergic neurons in the substantia nigra pars compacta (SNpc) and locus coeruleus (LC) and as the presence of cytoplasmic inclusions called Lewy bodies, mainly formed by fibrillar α-synuclein [86]. α-synuclein, a cytoplasmic protein, plays an important role in synaptic transmission and neuroplasticity [87]. α-synuclein has neuroprotective effects by regulating the synthesis of Dop, its storage into vesicles, its release in synapses and ultimately its reuptake into dopaminergic neurons [88]. However, α-synuclein expression was shown to be higher in both the blood and brain of PD patients than in those of age-matched controls [89,90]. It has been suggested that α-synuclein aggregation is concentration-dependent, so increased levels of α-synuclein may enhance the fibril formation of α-synuclein [89].

Melatonin was reported to inhibit oxidative stress and apoptosis by increasing the concentrations of Dop and preserving dopaminergic neurons in mouse models of PD induced by maneb and paraquat [91]. Other studies reported that melatonin alleviates oxidative stress, mitochondrial dysfunction and neurobehavioral deficits by increasing Dop levels in a 1-methyl-4-phenyl-1,2,3,6 tetrahydropyridine (MPTP)-induced mouse model of PD [92]. Several α-synuclein (SNCA) mutations, including A30P, have been found to contribute to sporadic PD [93]. Brito-Armas et al. showed that melatonin decreases the loss of dopaminergic neurons resulting from the SNCA mutant A30P [94].

Melatonin has been found to improve neurotoxicity by inhibiting autophagy and α-synuclein aggregation by enhancing the ubiquitination of α-synuclein in a kainic acid-induced mouse model [95]. It has also been reported that melatonin inhibits apoptosis induced by arsenite by blocking the aggregation of α-synuclein in rats [96]. Su et al. found that melatonin attenuates MPTP-induced autophagy and α-synuclein fibril formation by inhibiting CDK5 in monkeys [97]. Moreover, melatonin has been shown to attenuate amphetamine-induced neurotoxicity by reducing the expression of α-synuclein in vitro and in vivo [98]. Melatonin has also been found to reduce mitochondrial damage in a yeast model induced by expressing α-synuclein [99]. In addition, a reduction in the MT1 and MT2 levels in the amygdala and substantia nigra of the brain leads to PD [100]. However, Willis et al. reported that an imbalance of melatonin and Dop triggers PD [101]. Melatonin has been reported to decrease or inhibit the synthesis and release of Dop, and the loss of Dop is the cause of PD [102]. Moreover, these researchers showed that a melatonin receptor antagonist improves motor dysfunction in rats [103,104]. Therefore, more studies are needed to clarify these conflicting results.

### 5.3. Melatonin and Huntington’s Disease

Huntington’s disease (HD) is an autosomal dominant neurodegenerative disease triggered by an expanded cytosine–adenine–guanine (CAG) triplet in the gene encoding the Huntingtin (HTT) protein, which initially affects the striatum and cortex [105]. HD patients suffer from progressive motor decline (chorea, dystonia, dyskinesia and postural imbalance), cognitive impairment, psychiatric disorders (moodiness, severe anxiety and depression), sleep problems, dysphagia and weight loss [105,106]. HD is identified as intranuclear inclusions consisting of aggregated abnormal HTT in neuronal nuclei, cytoplasm, dendrites and axon terminals [106]. The aggregation of mutant HTT induces neuronal apoptosis, which may be caused by mitochondrial defects [107].

Melatonin levels are reduced in the plasma of HD patients compared with in healthy subjects [108]. Melatonin has protective effects against the neuronal cell death induced by kainic acid, which can lead to HD-like pathology in vitro and in vivo [109,110,111]. Melatonin inhibits the oxidative stress and neuronal damage generated from 3-nitropropionic acid (3-NP) exposure, which is used to mimic the pathology of HD in vitro and in vivo [112,113,114]. It has been reported that one of the major pathologies of HD is mitochondrial dysfunction [115]. Interestingly, Wang et al. showed that melatonin reduces neuronal cell death along with the preservation and activation of MT1 in an HTT mutant cell model [116]. Furthermore, the study showed that MT1 levels are lower in HD mice than in wild-type mice [116]. However, a nonselective melatonin receptor antagonist blocks the protective action of melatonin, which can alleviate mitochondrial dysfunction and prevent neuronal apoptosis [117]. Therefore, melatonin may reduce the neuronal cell death induced by mitochondrial defects in HD via an MT1-dependent pathway.

Another molecular mechanism for mitochondrial-dependent cell death in HD is the elevation of the intracellular Ca^2+^ concentration caused by its influx through the *N*-methyl-d-aspartate (NMDA) receptor channel [118,119]. It has been found that the induction of mitochondrial permeability transition pore (mPTP) results in cell death [120]. Andrabi et al. showed that melatonin diminishes the NMDA receptor-induced increase in Ca^2+^ by inhibiting mPTP activity in mouse primary striatal neurons [121].

In addition, melatonin has protective effects on mitochondria-induced neuronal apoptosis by regulating pro- or antiapoptotic proteins [122]. Mohseni et al. showed that melatonin protects peripheral blood lymphocytes in rats from gamma irradiation-induced apoptosis by increasing the levels of Bcl-2 and decreasing Bax expression and the Bax/Bcl-2 ratio [123]. According to Radogna et al., the antiapoptotic effect of melatonin is decreased by inhibiting Bcl-2 or by treating neurons with luzindole, an MT1/MT2 receptor antagonist. Therefore, melatonin attenuates cell death by inducing Bcl-2 expression and mitochondrial translocation through its interaction with MT1/MT2 receptors [124]. Furthermore, melatonin exerts its antiapoptotic function by promoting the interaction between Bcl-2 and Bax by binding to the MT1/MT2 receptors and by inducing the relocalization of the Bcl-2/Bax complex to the mitochondria through interactions with calmodulin [125].

### 5.4. Melatonin and Multiple Sclerosis

Multiple sclerosis (MS) is a progressive chronic inflammatory demyelinating disease of the central nervous system (CNS) [126,127]. MS is an immune-mediated disorder related to immune mediators, which mostly affects the white matter and gray matter of the CNS [128]. The clinical symptoms include numbness, weakness or even spastic paralysis in the limbs, pain, visual dysfunction, cognitive impairment, psychiatric disorders such as anxiety and depression, and sleep problems [129]. MS is the most common cause of nontraumatic disability among young adults [127]. The pathophysiology of MS is complex and associated with environmental factors, genetic factors and immune-mediated responses resulting in demyelination, axonal loss and neuroinflammation.

Abundant evidence has shown that melatonin levels in the serum, plasma and urine are lower in MS patients than those in control subjects [130,131,132]. However, other groups also found that melatonin levels are not changed in the serum between MS patients and healthy individuals [133]. Recently, melatonin was shown to exhibit antioxidative and anti-inflammatory effects during the demyelination and remyelination stages in a mouse model of MS induced by cuprizone [134]. Melatonin attenuates motor behavior deficits, including total distance moved (TDM) and velocity, during the demyelination stage in both male and female mice [134]. Moreover, melatonin reduces apoptosis by increasing Bcl-2 expression and decreasing caspase-3 and Bax levels, as well as through antioxidant activity by activating NF-κB and reducing heme oxygenase-1 expression [135]. Furthermore, melatonin increases the levels of antioxidative factors such as SOD, CAT, GSH and GPx and decreases malondialdehyde (MDA) levels, a marker of oxidative stress, during the demyelination stage [136]. In addition, melatonin has been discovered to exert an anti-inflammatory effect by reducing the levels of proinflammatory cytokines, including interleukin-1 beta (IL-1β) and TNF-α, during the demyelination stage [137,138,139]. Farez et al. revealed that melatonin decreases the levels of IL-17 secreted from TH17 cells via an MT1-dependent pathway [139]. The neuroprotective effects of melatonin during the demyelination stage were found in both males and females. However, the effects of melatonin during the remyelination stage were only observed in male mice but not in female mice [134]. Therefore, gender differences in the effects of melatonin during the remyelination stage remain to be elucidated in MS.

### 5.5. Melatonin and Amyotrophic Lateral Sclerosis

Amyotrophic lateral sclerosis (ALS) is characterized by the progressive and selective degeneration of motor neurons (MNs) in the brain stem, hypoglossal motor neurons (HMNs), facial motor neurons (FMNs) and the spinal cord, resulting in progressive paralysis and eventual death [140]. It has been reported that melatonin not only effectively delays the progression and mortality of the disease but also significantly inhibits motor neuron death by inactivating the receptor interacting protein-2 (Rip2)/caspase-1 pathway and caspase-3 and blocking the release of mitochondrial cytochrome c in a mutant superoxide dismutase 1 (SOD1) (G93A) transgenic mouse model of ALS [141]. The protective effect of melatonin on apoptosis in ALS was shown to be related to the inhibition of the caspase-1/cytochrome c/caspase-3 pathway. Moreover, it has also been shown that the levels of melatonin and MT1 but not MT2 are much lower in the spinal cord of ALS mice than those in wild-type mice [141]. Thus, the antiapoptotic effect of melatonin in ALS may be dependent on the MT1 pathway.

Rogério et al. found that melatonin reduces the loss of motor neurons in a neonatal rat model of peripheral nerve injury. Moreover, SOD1 expression is higher in the lumbar spinal cord in a model with the administration of melatonin compared with that without melatonin treatment [142]. Thus, melatonin may protect motor neurons from degeneration by elevating the expression of SOD1. It has been identified that neurofilaments accumulate in motor neurons in ALS patients as well as in SOD1 knockout mice [143,144]. Abnormal neurofilament accumulation could be either the cause or the consequence of neuronal degeneration [145]. Thus, melatonin eliminates neurofilament accumulation by increasing SOD1 expression, thereby preventing motor neuron loss.

Weishaupt et al. showed that melatonin reduces the cell death in cultured motor neurons induced by glutamate and inhibits the development and increases the survival of ALS in a mouse model (SOD1 (G93A)-transgenic mice). Interestingly, the researchers observed that the levels of circulating serum protein carbonyls, a marker for oxidative stress, are higher in ALS patients than those in healthy individuals. However, the concentrations of circulating serum protein carbonyls returned to normal levels in patients who were treated with melatonin. It has been suggested that melatonin attenuates neurodegeneration and the progression of ALS by inhibiting oxidative stress [41]. Estevez et al. found that mutations in SOD1, which are the major pathophysiological symptom of ALS, may decrease the antioxidant effect by affecting the binding ability of enzymes for zinc ions [146,147]. Moreover, zinc-deficient SOD1 induces motor neuronal apoptosis, which involves the endogenous production of nitric oxide (NO) [146]. Therefore, melatonin may exert antioxidant activity to prevent motor neuronal death through the suppression of NO formation. However, more studies are needed to further explore the molecular mechanism of the neuroprotective effects of melatonin on ALS.

### 5.6. Melatonin and Vascular Dementia

Vascular dementia (VD) is the second most common cause of dementia. Chronic cerebral hypoperfusion, which leads to hippocampal injury and cognitive impairment, is a major cause of VD [148,149]. VD has emerged as a major medical and health problem worldwide and imposes a mental and economic burden on individuals, families, communities and countries [150,151]. It has been reported that VD induced by chronic cerebral hypoperfusion has pathophysiological features, including oxidative stress, neuroinflammation and central cholinergic dysfunction [152,153]. Jaliah et al. showed the neuroprotective effects of melatonin on VD in a rat model generated by permanent bilateral common carotid artery occlusion (BCCAO) [154]. The data showed that melatonin increases the concentrations of acetylcholine (ACh), norepinephrine (NE) and Dop in the hippocampus [154]. The mechanism for the melatonin-induced elevation of ACh, NE and Dop levels depends on the uptake and release of the neurotransmitters NE and Dop by synaptosomes in the hypothalamus [155]. Moreover, melatonin effectively decreases oxidative stress markers, such as thiobarbituric acid reactive substances (TBARS), and increases the levels of antioxidative factors, including SOD, CAT, glutathione (GSH) and total antioxidant capacity (TAC) [154]. The induction of TBARS and reduction of SOD, CAT, GSH and TAC are due to ROS overload, which induces oxidative damage to DNA, RNA, protein and lipids and finally leads to neuronal impairment and cell death [156]. However, melatonin has antioxidant effects on motor neurons, attenuating the production of ROS, mediated by melatonin receptors [152,157,158]. Furthermore, melatonin has anti-inflammatory activity, further limiting the production of excessive amounts of ROS [158,159]. Melatonin produces antioxidant and anti-inflammatory effects through the modulation of JNK, NF-κB, hypoxia-inducible factor-1 alpha (HIF-1α), nuclear factor erythroid 2-related factor 2 (Nrf2) and others [158,159]. In addition, Jaliah et al. discovered that melatonin significantly increases the expression of senescence marker protein-30 (SMP30) and osteopontin (OPN) [154]. SMP30 suppresses apoptosis through the modulation of Ca^2+^-pump activity in the cell membrane [160]. Melatonin has also been found to inhibit autophagy by restoring the expression of SMP30 [161]. OPN is neuroprotective due to its role in downregulating inducible nitric oxide synthase (iNOS) and increasing NF-κB and PI3K activity [162,163]. OPN decreases the generation of ROS and eventually leads to antiapoptotic effects by suppressing cleaved caspase-3 [164]. Other studies also showed that melatonin increases the expression of OPN and inhibits apoptosis via the upregulation of Bcl-2 and downregulation of Bax in the hippocampus [62,154,165,166].

Shen et al. found that melatonin improves cognitive dysfunction along with the suppression of oxidative stress, neuroinflammation, brain-derived neurotrophic factor (BDNF) depletion and central cholinergic dysfunction in a rat model of VD induced by BCCAO [167]. BDNF plays a critical role in the regulation of neuroprotection, neuroregeneration and synaptic plasticity as well as cognitive and affective behaviors [168,169]. Melatonin has been shown to exert neuroprotective effects by elevating the level of BDNF [170]. Further studies are needed to clarify the mechanism of melatonin in protecting against central cholinergic dysfunction.

## 6. Clinical Application of Melatonin in Neurodegenerative Diseases

Melatonin has been investigated for its effects on the clinical symptoms of neurodegenerative diseases because of its beneficial effects on multiple experimental cell and animal models. The details of both case reports and clinical trials of melatonin supplementation in AD patients are summarized in Table 7. The first case report was conducted in a pair of twins with AD, which showed that melatonin treatment improved cognitive dysfunction, behavioral problems, sundown syndrome and sleep quality during melatonin treatment [171]. Sundown syndrome or sundowning, which is a neuropsychiatric phenomenon appearing in the late afternoon or early evening, is commonly identified in AD patients [172,173]. Another case report, on a man with typical sundown syndrome, showed improvements in sleep and behavior with a dose of 2 mg of melatonin treatment at 8 p.m. for one week and gradual improvement with an extra dose of 2 mg at 3 p.m. for two weeks [174]. Moreover, melatonin reduces the incidence of rapid eye movement (REM), a kind of sleep behavior [175]. However, other studies showed that melatonin may not improve the symptoms for all AD patients. Melatonin was found to ameliorate the circadian rest–activity rhythm, sleepy state during the daytime and mood in one patient. However, melatonin only ameliorated cognitive exacerbation but did not improve any other symptoms in the other patient [176].

In addition to case reports, clinical trials have been performed to explore the effects of melatonin on clinical symptoms in AD patients. A study including 80 patients revealed improvements in sleep problems and cognitive impairments after melatonin treatment, in which nocturnal sleep was evaluated subjectively by the Pittsburg Sleep Quality Index (PSQI) and diaries [177]. However, Serfaty et al. and Alves et al. failed to show beneficial effects of melatonin on sleep quality or cognitive functions after the administration of melatonin for 2 weeks and 10 days, respectively [178,179]. The negative results may be due to the short-term melatonin treatment and the method of analyzing sleep parameters. However, the sleep and cognitive disturbances of the AD patients were not attenuated by melatonin treatment for 2 months with the same objective method of assessing sleep efficiency in another report [180]. Nevertheless, in this report, a slight improvement in sleep quality was discovered by the subjective measurement (PSQI) for sleep efficiency [180]. It is suggested that it is necessary to have subjective sleep measurements, not only the objective methods, because subjective methods are more stable. However, another randomized and double-blind trial performed in 20 AD patients suggested an improvement in sleep quality after melatonin treatment, with sleep measured objectively by actigraphy [181].

There are many other clinical investigations, which provide evidence to support the beneficial effects of melatonin on sleep disorders and cognitive deficits [6,52,182,183,184,185,186,187,188,189,190,191]. In addition, the combination of bright light exposure and melatonin supplementation has been discovered to improve circadian rest–activity or sleep–wake rhythms, sundowning symptoms and sleep quality in AD patients [192,193]. Although melatonin has been reported to stabilize circadian rhythms, reduce daytime sleepiness, improve sleep quality and delay the progression of cognitive impairment in most case reports and clinical trials, some AD patients experience little or no benefits after the administration of melatonin in some clinical studies. Therefore, further studies are necessary to confirm the efficacy of melatonin for clinical symptoms in AD patients. Moreover, other adjuvant therapies that can be used together with melatonin supplementation are of interest.

Variations in the endogenous levels of melatonin have been detected in PD patients receiving melatonin supplementation [194]. Clinical studies have been conducted to explore the effect of exogenous melatonin on clinical symptoms in PD patients [6,190,191]. As summarized in Table 7, two double-blind and placebo-controlled clinical studies showed an improvement in sleep disturbances in patients with PD following melatonin treatment [195,196]. Dowling et al. performed a clinical trial in 40 PD patients who received melatonin for 2 weeks. Patients taking 50 mg of melatonin at bedtime showed significant improvements in sleep quality, and the sleep parameters were measured by actigraphy, but not in those receiving 5 mg every day [195]. This finding is consistent with the pilot study in which some patients with PD experienced no significant improvement in sleep quality or quantity at night after the administration of 5 mg of melatonin for 1 week [197]. Nevertheless, melatonin has been found to significantly alleviate sleep disorders, sleep quantity and daytime somnolence according to a subjectively evaluated method in patients with 5 mg of melatonin [195]. It is suggested that not only PD patients but also healthy elderly individuals take a high dose of melatonin over a 10-week period. Medeiros et al. reported that melatonin efficiently attenuated sleep disturbances according to subjective measurements but not according to polysomnography in PD patients treated with melatonin at 3 mg/day for 4 weeks [196]. However, melatonin failed to improve motor dysfunctions in this investigation, which may be due to the small sample size and the low sensitivity of the method for assessing motor parameters. In addition, a randomized controlled trial with 38 PD patients discovered that melatonin supplementation of 3 mg for 6 weeks led to the reduction of wake time at night and daytime sleepiness, according to both an objective method and subjective evaluations, as well as an improvement in cognitive functions [198]. In a recent study, melatonin was discovered to significantly enhance sleep quality and reduce anxiety in patients with early or late stages of PD, while no significant alterations in autonomic disorder, cognitive dysfunction, motor impairment or depression status were observed after melatonin supplementation [199]. These negative studies suggest that it is important to design suitable parameters, such as the dose and time of treatment and the methods for evaluating sleep quality, cognitive function or motor status.

Most of the clinical studies on the use of melatonin to attenuate clinical symptoms in PD found that melatonin treatment reduced nonmotor disorders, especially the incidence of sleep disturbances and excessive daytime somnolence, but no improvement in motor deficits was observed. Furthermore, current evidence is insufficient to support the use of melatonin for the prevention or treatment of clinical manifestations in PD. Therefore, more clinical studies are necessary to confirm the beneficial effects of melatonin on human PD.

A limited number of clinical studies were carried out to investigate the efficacy of melatonin in other neurodegenerative disorders, such as ALS and MS. The first melatonin therapy for ALS was conducted in three subjects with 30–60 mg of slow-release melatonin orally at night for 13 months. The patient with the latest stage of ALS was shown to have an attenuated progression of ALS after melatonin administration, whereas two patients showed reduced deterioration at the last test [200]. Another clinical study was performed in 31 ALS patients receiving 30 mg/day of melatonin at bedtime for 24 months as an adjuvant therapy. In this study, it was concluded that high-dose melatonin is suitable for clinical trials to reduce oxidative stress in ALS, rather than having neuroprotective effects on clinical symptoms in ALS [41]. The current data are insufficient to draw a definitive conclusion on the efficacy of melatonin in the treatment of ALS. There are also studies to discover whether melatonin influences the quality of life of patients with MS. Combinations of melatonin, interferon-beta and glatiramer acetate were found to improve the quality of life of MS patients taking 5 mg/day of melatonin for 3 months in a controlled clinical trial [201]. Moreover, a few other clinical trials on melatonin intervention have been carried out to examine the efficacy and safety of melatonin administration in MS patients. Some of them have completed or terminated, while others are recruiting now or have not yet started. The role of melatonin in the treatment of MS needs to be explored further in more clinical studies [202].

## 7. Conclusions

Melatonin plays a critical role in the improvement of circadian rhythms, oxidative stress, inflammatory activity, neuronal loss, mitochondrial impairment and clinical symptoms through melatonin receptor-dependent or melatonin receptor-independent pathways in a variety of neurodegenerative diseases, such as AD, PD, HD, ALS, VD and MS. Nevertheless, more experimental cell and animal models are required for a better understanding of the molecular mechanisms to attenuate the sleep, motor and nonmotor dysfunctions of the patients. Moreover, clinical studies have shown that melatonin is a useful and competent therapeutic tool in neurodegenerative disorders. However, most studies have focused on the sleep-promoting effects as well as the suppression of sundown syndrome and cognitive deficits. Moreover, clinical trials in PD, HD, ALS, VD and MS are still limited. The quality of clinical studies varies because of the different formulations, doses and durations of melatonin treatment, as well as the different methods in the study designs and behavioral evaluations. Thus, well-designed, large multicenter clinical trials are urgently needed to further investigate the potential and usefulness of melatonin for the clinical symptoms of neurodegenerative patients.

Although melatonin displays almost no side effects, even with high-dose and long-term administration, in patients in most clinical studies, some adverse reactions of melatonin—including drowsiness, fever, headache, vomiting, thrombosis, drowsiness, hyperkinesia or restless leg syndrome—may occur [10,203]. Therefore, the use of melatonin should be considered seriously with respect to the dosage and duration. Presently, some synthetic melatonergic drugs, such as ramelteon, agomelatine, tasimelteon and TK-301, are being used in the clinic to reduce sleep latency [25,191]. Regarding the short half-life (less than 30 min), which may be one of the reasons for the inconsistent results reported in clinical trials, the development of melatonin with a prolonged release is needed [191,204]. Melatonin controlled-release tablets (Circadin, Neurim, Tel-Aviv, Israel) may overcome this issue [25,191]. Several other compounds are being investigated to obtain selective and effective activities of melatonin in clinical dysfunctions, thereby providing a promising future for the therapy of neurodegenerative diseases by the use of melatonin [205,206].

## Figures and Tables

**Table 1:** Melatonin and Alzheimer’s disease.

Effects	Model	Signaling Pathway	Concentrations	Reference
Inhibiting apoptosis	Cell	Bax/bcl-2/caspase-3	Pharma	[21]
Inhibiting Aβ neurotoxicity	Cell	Pin1/GSK3β/NF-κB	Physio	[57]
Inhibiting amyloid fibrils	Cell	Apoe4	Pharma	[59]
Inhibiting apoptosis	Animal	Bax/caspase-3/Par-4	Pharma	[60]
Inhibiting tau hyperphosphorylation	Cell	GSK-3β	Pharma	[61]
Inhibiting tau hyperphosphorylation	Animal	PI3K/Akt/GSK3β	Pharma	[62]
Inhibiting phosphorylation and accumulation of neurofilaments	Cell	PP-2A/PP-1	Pharma	[67]
Inhibiting tau hyperphosphorylation	Animal	PP-2A/PP-1	Pharma	[68]
Inhibiting tau hyperphosphorylation	Animal	GSK3β/PKA/ PP-2A/PP-1 and ER Stress	Pharma	[69]
Inhibiting tau hyperphosphorylation	Cell and Animal	ER Stress/GSK-3β/CDK5	Pharma	[70]
Inhibiting tau hyperphosphorylation	Animal	PKA	Pharma	[71]
Inhibiting tau hyperphosphorylation and oxidative stress	Cell	GSK-3β	Pharma	[72]
Inhibiting tau hyperphosphorylation and Aβ neurotoxicity	Animal	GSK-3β	Pharma	[74]
Inhibiting phosphorylation of neurofilaments	Animal	CDK5	Pharma	[75]
Regulating circadian rhythms	Cell	PKC	Physio	[76]
Inhibiting tau hyperphosphorylation and oxidative stress	Cell	PP-2A/GSK-3β	Pharma	[77]
Inhibiting tau hyperphosphorylation	Animal	PP-2A	Pharma	[78]
Inhibiting apoptosis	Cell	Calpain/CDK5	Pharma	[79]
Inhibiting tau hyperphosphorylation	Cell	DAPK1/Pin1	Physio	[80]

Abbreviations: Pharma, pharmacological concentration; Physio, physiological concentration.

**Table 2:** Melatonin and Parkinson’s disease.

Effects	Model	Signaling Pathway	Concentrations	Reference
Inhibiting apoptosis and oxidative stress	Animal	CYP2E1/GST/p53/Bax/caspase-9	Pharma	[91]
Inhibiting autophagy and α-synuclein aggregation	Animal	Caspase-3/12 and LC3-II/LAMP-2/cathepsin B	Pharma	[95]
Inhibiting apoptosis	Animal	ER stress/Bcl2/caspase-3	Pharma	[96]
Inhibiting autophagy and α-synuclein	Cell	CDK5	Pharma	[97]

Abbreviations: Pharma, pharmacological concentration; Physio, physiological concentration.

**Table 3:** Melatonin and Huntington’s disease.

Effects	Model	Signaling Pathway	Concentrations	Reference
Inhibiting apoptosis	Animal	ER Stress	Pharma	[109]
Inhibiting cell death	Cell and Animal	MT1 receptor	Pharma	[116]
Improving mitochondrial dysfunction	Animal	Apelin 13	Pharma	[117]
Inhibiting apoptosis	Animal	Caspase-3	Pharma	[121]
Inhibiting apoptosis	Animal	Bax/bcl-2	Pharma	[123]
Inhibiting apoptosis	Cell	Bax/bcl-2	Pharma	[124]
Inhibiting apoptosis	Cell	Bax/bcl-2	Physio and Pharma	[125]

Abbreviations: Pharma, pharmacological concentration; Physio, physiological concentration.

**Table 4:** Melatonin and multiple sclerosis.

Effects	Model	Signaling Pathway	Concentrations	Reference
Inhibiting apoptosis	Animal	NF-κB/bax/bcl-2	Pharma	[135]
Anti-inflammatory	Animal	MT1/Erk1/2	Pharma	[139]

Abbreviations: Pharma, pharmacological concentration; Physio, physiological concentration.

**Table 5:** Melatonin and amyotrophic lateral sclerosis.

Effects	Model	Signaling Pathway	Concentrations	Reference
Inhibiting apoptosis	Animal	Caspase-1/cytochrome c/caspase-3	Pharma	[141]
Inhibiting oxidative stress	Animal	SOD1/SOD2/nNOS	Pharma	[142]

Abbreviations: Pharma, pharmacological concentration; Physio, physiological concentration.

**Table 6:** Melatonin and vascular dementia.

Effects	Model	Signaling Pathway	Concentrations	Reference
Inhibiting oxidative stress	Animal	SMP30/OPN	Pharma	[154]
Inhibiting oxidative stress	Cell	MT1/MT2	Physio	[157]
Inhibiting oxidative stress	Animal	RAGE/NF-κB/JNK	Pharma	[158]
Inhibiting autophagy	Cell	MTOR	Pharma	[161]
Inhibiting apoptosis	Animal	SIRT1/bax/bcl-2	Pharma	[165]

Abbreviations: Pharma, pharmacological concentration; Physio, physiological concentration.

**Table 7:** Clinical studies including the treatment of AD and PD patients with melatonin.

Design	Subjects	Treatment	Assessment	Results	Reference
CR	2 AD patients (age: 79 years)	6 mg at bedtime for 36 months	Cognitive evaluation by FAST; neuroimaging evaluation by NMR	Significant improvement of sleep quality, reduction of sundowning, and lack of progression of cognitive and behavioral disorders	[171]
CR	1 AD patient (age: 81 years)	2 mg at 8 p.m. for 1 week, 2 mg at 3 p.m. and 8 p.m. for 2 weeks	Cognitive evaluation by MMSE; neuropsychiatric evaluation by NPI	Significant improvement of sleep quality and behavioral symptoms after the first week, and gradual improvement over the subsequent two weeks	[174]
CR	1 AD patient (age: 68 years)	5–10 mg at bedtime for 20 months	Sleep evaluation by PSG	Significant effects on suppression of REM sleep behavior disorder	[175]
CR	2 AD patients (age: 72 and 75 years)	6 mg (2 h before bedtime) for 35 days	Sleep evaluation by actigraphy; cognitive evaluation by ADAS and MMSE	Significant improvement of the circadian rest–activity rhythm and mood and reduction of daytime sleepiness in one of them	[176]
R, DB, PC	73 AD patients (mean age: 75.3 years)	2 mg (slow-release, 1–2 h before bedtime) for 24 weeks	Sleep evaluation by PSQI; cognitive evaluation by ADAS, MMSE and IADL	Significant improvement of sleep efficiency and cognitive performance	[177]
R, DB, PC	41 AD patients (age: 61–95 years)	1.5 mg (slow-release) and 8.5 mg (fast-release) at 10 p.m. for 10 days	Sleep evaluation by actigraphy	No significant effects on sleep, circadian rhythms or agitated behaviors	[178]
R, DB, PC	25 patients with dementia (21 AD patients, age: over 65 years)	6 mg (slow-release) at bedtime for 2 weeks	Sleep evaluation by actigraphy; cognitive evaluation by MMSE	No significant effects on sleep or cognitive function	[179]
R, DB, PC	20 AD patients (mean age: 79.2 years)	3 mg at 8.5 p.m. for 4 weeks	Sleep evaluation by actigraphy; cognitive evaluation by ADAS, MMSE and CDRS	Significant improvement of the sleep–wake rhythm, cognitive dysfunction and behavioral problems	[181]
R, PC	157 AD patients (mean age: 77.4 years)	2.5 mg (slow-release) or 10 mg (fast-release),1 h before bedtime for 2 months	Sleep evaluation by actigraphy and diary; cognitive evaluation by ADAS, MMSE and IADL; neuropsychiatric evaluation by NPI and SDI	No significant effects on sleep disturbances by actigraphy; slightly improvement of sleep quality by diary; no effects on cognitive function	[180]
R, PC	24 AD patients (mean age: 78.6 years)	3 mg at bedtime for 2 weeks	Sleep evaluation by actigraphy; neuropsychiatric evaluation by NPI	Significant improvement of circadian rhythm disturbances, agitation and behavioral symptoms	[186]
R, PC	50 AD patients (mean age: 86 years)	5 mg melatonin and 1 h morning light (≥2500 lux) for 10 weeks	Sleep evaluation by actigraphy	Significant improvement of the rest–activity rhythm	[192]
OL	14 AD patients (mean age: 72 years)	9 mg at bedtime for 22–35 months	Sleep evaluation by diary; cognitive evaluation by FAST, ADAS, MMSE and Mattis’ and Blessed’s scales	Significant improvement of sleep quality; no cognitive or behavioral deterioration and loss of sundown syndrome	[171]
OL	10 AD patients (mean age: 74 years)	3 mg at bedtime for 3 weeks	Sleep evaluation by diary	Significant improvement of sleep disturbances and sundowning	[182]
OL	11 AD patients (mean age: 85 years)	3 mg at bedtime for 3 weeks	Sleep evaluation by diary	Significant attenuation of daytime sleepiness and agitation	[183]
OL, PC	14 AD patients	6 mg at 9 p.m. for 4 weeks	Sleep evaluation by actigraphy and diary	Significant improvement of insomnia	[184]
OL	45 AD patients (mean age: 73 years)	6–9 mg at bedtime for 4 months	Sleep evaluation by diary; cognitive evaluation by FAST	Significant improvement of sleep quality, sundowning, and cognitive and behavioral impairment	[185]
OL	7 AD patients (mean age: 75.6 years)	3 mg at around 9 p.m. for 3 weeks	Sleep evaluation by actigraphy; cognitive evaluation by MMSE and GDS	Significant improvement of circadian rhythm dysfunction and sundown syndrome	[51]
R, DB, PC	40 PD patients (age: 40–80 years)	5–50 mg at bedtime for 2 weeks	Sleep evaluation by actigraphy and diary, ESS, SSS and GSDS	Significant increased nighttime sleep with 50 mg by objective; significant improvement of sleep quality with 5 mg only by subjective but not objective	[195]
R, DB, PC	18 PD patients (mean age: 61.8 years)	3 mg at bedtime for 4 weeks	Sleep evaluation by PSG, PSQI and ESS; motor evaluation by UPDRS	Significant improvement of sleep quality; no improvement of motor dysfunction	[196]
R	38 PD patients (mean age: 67.3 years)	3 mg (30 min before bedtime) for 6 weeks	Sleep evaluation by PSG, PDSS and ESS; cognitive evaluation by MMSE, five-word test, digit span and the Hamilton scale	Significant improvement of sleep quality, daytime sleepiness and cognitive dysfunction	[198]
R	30 PD patients (mean age: 64.1 years)	3 mg at bedtime for 2 months	Sleep evaluation by PDSS and ESS; neuropsychiatric evaluation by Beck’s scale and Spielberger’s scale	Significant improvement of sleep quality and anxiety status; no significant changes in motor, cognitive or autonomic dysfunction or depression status	[199]

Abbreviations: CR: Case report; R: Randomized; DB: Double-blind; PC: Placebo-controlled; OL: Open-label; FAST: Functional Assessment Tool for Alzheimer Disease; NMR: Nuclear Magnetic Resonance; MMSE: Mini–Mental State Examination; NPI: Neuropsychiatric Inventory; PSG: Polysomnography; ADAS: Alzheimer’s Disease Assessment Scale; PSQI: Pittsburgh Sleep Quality Index; IADL: Instrumental Activities of Daily Living; CDRS: Clinical Dementia Rating Scale; SDI: Sleep Disorders Inventory; GDS: Global Deterioration Scale; ESS: Epworth Sleepiness Scale; SSS: Stanford Sleepiness Scale; GSDS: General Sleep Disturbance Scale; UPDRS: Unified Parkinson’s Disease Rating Scale; PDSS: Parkinson’s Disease Sleep Scale.

## References

[1] Lerner A.B., Case J.D., Takahashi Y., Lee T.H., Mori W. (1958). Isolation of melatonin, the pineal gland factor that lightens melanocytes1. J. Am. Chem. Soc..

[2] Claustrat B., Brun J., Chazot G. (2005). The basic physiology and pathophysiology of melatonin. Sleep Med. Rev..

[3] Slats D., Claassen J.A., Verbeek M.M., Overeem S. (2013). Reciprocal interactions between sleep, circadian rhythms and alzheimer’s disease: Focus on the role of hypocretin and melatonin. Ageing Res. Rev..

[4] Vriend J., Reiter R.J. (2015). Melatonin feedback on clock genes: A theory involving the proteasome. J. Pineal Res..

[5] Reiter R.J., Rosales-Corral S.A., Tan D.X., Acuna-Castroviejo D., Qin L., Yang S.F., Xu K. (2017). Melatonin, a full service anti-cancer agent: Inhibition of initiation, progression and metastasis. Int. J. Mol. Sci..

[6] Cardinali D.P. (2019). Melatonin: Clinical perspectives in neurodegeneration. Front. Endocrinol..

[7] Zhang R., Wang X., Ni L., Di X., Ma B., Niu S., Liu C., Reiter R.J. (2020). Covid-19: Melatonin as a potential adjuvant treatment. Life Sci..

[8] Talib W.H. (2018). Melatonin and cancer hallmarks. Molecules.

[9] Gan L., Cookson M.R., Petrucelli L., La Spada A.R. (2018). Converging pathways in neurodegeneration, from genetics to mechanisms. Nat. Neurosci..

[10] Gunata M., Parlakpinar H., Acet H.A. (2020). Melatonin: A review of its potential functions and effects on neurological diseases. Revue Neurol..

[11] Reiter R.J. (1991). Pineal melatonin: Cell biology of its synthesis and of its physiological interactions. Endocr. Rev..

[12] Stokkan K.A., Reiter R.J. (1994). Melatonin rhythms in arctic urban residents. J. Pineal Res..

[13] Benot S., Molinero P., Soutto M., Goberna R., Guerrero J.M. (1998). Circadian variations in the rat serum total antioxidant status: Correlation with melatonin levels. J. Pineal Res..

[14] He R., Cui M., Lin H., Zhao L., Wang J., Chen S., Shao Z. (2018). Melatonin resists oxidative stress-induced apoptosis in nucleus pulposus cells. Life Sci..

[15] Reiter R.J., Tan D.X., Erren T.C., Fuentes-Broto L., Paredes S.D. (2009). Light-mediated perturbations of circadian timing and cancer risk: A mechanistic analysis. Integr. Cancer Ther..

[16] Gupta M., Gupta Y.K., Agarwal S., Aneja S., Kalaivani M., Kohli K. (2004). Effects of add-on melatonin administration on antioxidant enzymes in children with epilepsy taking carbamazepine monotherapy: A randomized, double-blind, placebo-controlled trial. Epilepsia.

[17] Rodriguez C., Mayo J.C., Sainz R.M., Antolín I., Herrera F., Martín V., Reiter R.J. (2004). Regulation of antioxidant enzymes: A significant role for melatonin. J. Pineal Res..

[18] Hirata F., Hayaishi O., Tokuyama T., Seno S. (1974). In vitro and in vivo formation of two new metabolites of melatonin. J. Biol. Chem..

[19] Rosales-Corral S., Tan D.X., Reiter R.J., Valdivia-Velázquez M., Martínez-Barboza G., Acosta-Martínez J.P., Ortiz G.G. (2003). Orally administered melatonin reduces oxidative stress and proinflammatory cytokines induced by amyloid-beta peptide in rat brain: A comparative, in vivo study versus vitamin c and e. J. Pineal Res..

[20] Yokota O., Terada S., Ishizu H., Ishihara T., Ujike H., Nakashima H., Nakashima Y., Kugo A., Checler F., Kuroda S. (2003). Cyclooxygenase-2 in the hippocampus is up-regulated in alzheimer’s disease but not in variant alzheimer’s disease with cotton wool plaques in humans. Neurosci. Lett..

[21] Jang M.H., Jung S.B., Lee M.H., Kim C.J., Oh Y.T., Kang I., Kim J., Kim E.H. (2005). Melatonin attenuates amyloid beta25-35-induced apoptosis in mouse microglial bv2 cells. Neurosci. Lett..

[22] Wang J., Xiao X., Zhang Y., Shi D., Chen W., Fu L., Liu L., Xie F., Kang T., Huang W. (2012). Simultaneous modulation of cox-2, p300, akt, and apaf-1 signaling by melatonin to inhibit proliferation and induce apoptosis in breast cancer cells. J. Pineal Res..

[23] Menéndez-Menéndez J., Hermida-Prado F. (2019). Deciphering the molecular basis of melatonin protective effects on breast cells treated with doxorubicin: Twist1 a transcription factor involved in emt and metastasis, a novel target of melatonin. Cancers.

[24] Ekmekcioglu C. (2006). Melatonin receptors in humans: Biological role and clinical relevance. Biomed. Pharmacother..

[25] Emet M., Ozcan H., Yayla M., Halici Z., Hacimuftuoglu A. (2016). A review of melatonin, its receptors and drugs. Eurasian J. Med..

[26] Pandi-Perumal S.R., Trakht I., Srinivasan V., Spence D.W., Maestroni G.J., Zisapel N., Cardinali D.P. (2008). Physiological effects of melatonin: Role of melatonin receptors and signal transduction pathways. Prog. Neurobiol..

[27] Comai S., Gobbi G. (2014). Unveiling the role of melatonin mt2 receptors in sleep, anxiety and other neuropsychiatric diseases: A novel target in psychopharmacology. J. Psychiatry Neurosci. JPN.

[28] Nosjean O., Ferro M., Coge F., Beauverger P., Henlin J.M., Lefoulon F., Fauchere J.L., Delagrange P., Canet E., Boutin J.A. (2000). Identification of the melatonin-binding site mt3 as the quinone reductase 2. J. Biol. Chem..

[29] Carlberg C. (2000). Gene regulation by melatonin. Ann. N. Y. Acad. Sci..

[30] Garcia J.A., Volt H., Venegas C., Doerrier C., Escames G., Lopez L.C., Acuna-Castroviejo D. (2015). Disruption of the nf-kappab/nlrp3 connection by melatonin requires retinoid-related orphan receptor-alpha and blocks the septic response in mice. FASEB J..

[31] Cardinali D.P., Freire F. (1975). Melatonin effects on brain. Interaction with microtubule protein, inhibition of fast axoplasmic flow and induction of crystaloid and tubular formations in the hypothalamus. Mol. Cell. Endocrinol..

[32] Melendez J., Maldonado V., Ortega A. (1996). Effect of melatonin on beta-tubulin and map2 expression in nie-115 cells. Neurochem. Res..

[33] Macias M., Escames G., Leon J., Coto A., Sbihi Y., Osuna A., Acuna-Castroviejo D. (2003). Calreticulin-melatonin. An unexpected relationship. Eur. J. Biochem..

[34] Benitez-King G., Anton-Tay F. (1993). Calmodulin mediates melatonin cytoskeletal effects. Experientia.

[35] Benitez-King G., Huerto-Delgadillo L., Anton-Tay F. (1991). Melatonin modifies calmodulin cell levels in mdck and n1e-115 cell lines and inhibits phosphodiesterase activity in vitro. Brain Res..

[36] Benitez-King G., Rios A., Martinez A., Anton-Tay F. (1996). In vitro inhibition of ca2+/calmodulin-dependent kinase ii activity by melatonin. Biochim. Biophys. Acta.

[37] Jenwitheesuk A., Nopparat C., Mukda S., Wongchitrat P., Govitrapong P. (2014). Melatonin regulates aging and neurodegeneration through energy metabolism, epigenetics, autophagy and circadian rhythm pathways. Int. J. Mol. Sci..

[38] Reiter R.J., Tan D.X., Manchester L.C., Pilar Terron M., Flores L.J., Koppisepi S. (2007). Medical implications of melatonin: Receptor-mediated and receptor-independent actions. Adv. Med. Sci..

[39] Reiter R.J. (1995). The pineal gland and melatonin in relation to aging: A summary of the theories and of the data. Exp. Gerontol..

[40] Reiter R.J., Tan D.X., Poeggeler B., Menendez-Pelaez A., Chen L.D., Saarela S. (1994). Melatonin as a free radical scavenger: Implications for aging and age-related diseases. Ann. N. Y. Acad. Sci..

[41] Weishaupt J.H., Bartels C., Polking E., Dietrich J., Rohde G., Poeggeler B., Mertens N., Sperling S., Bohn M., Huther G. (2006). Reduced oxidative damage in als by high-dose enteral melatonin treatment. J. Pineal Res..

[42] Reiter R.J., Tan D.X., Sainz R.M., Mayo J.C., Lopez-Burillo S. (2002). Melatonin: Reducing the toxicity and increasing the efficacy of drugs. J. Pharm. Pharmacol..

[43] Ballatore C., Lee V.M., Trojanowski J.Q. (2007). Tau-mediated neurodegeneration in alzheimer’s disease and related disorders. Nat. Rev. Neurosci..

[44] Hardy J., Selkoe D.J. (2002). The amyloid hypothesis of alzheimer’s disease: Progress and problems on the road to therapeutics. Science.

[45] Jack C.R.J., Holtzman D.M. (2013). Biomarker modeling of alzheimer’s disease. Neuron.

[46] Polanco J.C., Li C., Bodea L.G., Martinez-Marmol R., Meunier F.A., Gotz J. (2018). Amyloid-beta and tau complexity-towards improved biomarkers and targeted therapies. Nat. Rev. Neurol..

[47] Spires-Jones T.L., Hyman B.T. (2014). The intersection of amyloid beta and tau at synapses in alzheimer’s disease. Neuron.

[48] Wu Y.H., Swaab D.F. (2005). The human pineal gland and melatonin in aging and alzheimer’s disease. J. Pineal Res..

[49] Wu Y.H., Feenstra M.G., Zhou J.N., Liu R.Y., Torano J.S., Van Kan H.J., Fischer D.F., Ravid R., Swaab D.F. (2003). Molecular changes underlying reduced pineal melatonin levels in alzheimer disease: Alterations in preclinical and clinical stages. J. Clin. Endocrinol. Metab..

[50] Zhou J.N., Liu R.Y., Kamphorst W., Hofman M.A., Swaab D.F. (2003). Early neuropathological alzheimer’s changes in aged individuals are accompanied by decreased cerebrospinal fluid melatonin levels. J. Pineal Res..

[51] Mahlberg R., Kunz D., Sutej I., Kuhl K.P., Hellweg R. (2004). Melatonin treatment of day-night rhythm disturbances and sundowning in alzheimer disease: An open-label pilot study using actigraphy. J. Clin. Psychopharmacol..

[52] Brusco L.I., Marquez M., Cardinali D.P. (2000). Melatonin treatment stabilizes chronobiologic and cognitive symptoms in alzheimer’s disease. Neuro Endocrinol. Lett..

[53] Zhang T., Chen D., Lee T.H. (2020). Phosphorylation signaling in app processing in alzheimer’s disease. Int. J. Mol. Sci..

[54] Lahiri D.K. (1999). Melatonin affects the metabolism of the beta-amyloid precursor protein in different cell types. J. Pineal Res..

[55] Wang X.C., Zhang Y.C., Chatterjie N., Grundke-Iqbal I., Iqbal K., Wang J.Z. (2008). Effect of melatonin and melatonylvalpromide on beta-amyloid and neurofilaments in n2a cells. Neurochem. Res..

[56] Zhang Y.C., Wang Z.F., Wang Q., Wang Y.P., Wang J.Z. (2004). Melatonin attenuates beta-amyloid-induced inhibition of neurofilament expression. Acta Pharmacol. Sin..

[57] Chinchalongporn V., Shukla M., Govitrapong P. (2018). Melatonin ameliorates abeta42 -induced alteration of betaapp-processing secretases via the melatonin receptor through the pin1/gsk3beta/nf-kappab pathway in sh-sy5y cells. J. Pineal Res..

[58] Pappolla M., Bozner P., Soto C., Shao H., Robakis N.K., Zagorski M., Frangione B., Ghiso J. (1998). Inhibition of alzheimer beta-fibrillogenesis by melatonin. J. Biol. Chem..

[59] Poeggeler B., Miravalle L., Zagorski M.G., Wisniewski T., Chyan Y.J., Zhang Y., Shao H., Bryant-Thomas T., Vidal R., Frangione B. (2001). Melatonin reverses the profibrillogenic activity of apolipoprotein e4 on the alzheimer amyloid abeta peptide. Biochemistry.

[60] Feng Z., Zhang J.T. (2005). Long-term melatonin or 17beta-estradiol supplementation alleviates oxidative stress in ovariectomized adult rats. Free Radic. Biol. Med..

[61] Hoppe J.B., Frozza R.L., Horn A.P., Comiran R.A., Bernardi A., Campos M.M., Battastini A.M., Salbego C. (2010). Amyloid-beta neurotoxicity in organotypic culture is attenuated by melatonin: Involvement of gsk-3beta, tau and neuroinflammation. J. Pineal Res..

[62] Ali T., Kim M.O. (2015). Melatonin ameliorates amyloid beta-induced memory deficits, tau hyperphosphorylation and neurodegeneration via pi3/akt/gsk3β pathway in the mouse hippocampus. J. Pineal Res..

[63] Peineau S., Taghibiglou C., Bradley C., Wong T.P., Liu L., Lu J., Lo E., Wu D., Saule E., Bouschet T. (2007). Ltp inhibits ltd in the hippocampus via regulation of gsk3beta. Neuron.

[64] Jo J., Whitcomb D.J., Olsen K.M., Kerrigan T.L., Lo S.C., Bru-Mercier G., Dickinson B., Scullion S., Sheng M., Collingridge G. (2011). Aβ(1-42) inhibition of ltp is mediated by a signaling pathway involving caspase-3, akt1 and gsk-3β. Nat. Neurosci..

[65] Lee K.Y., Koh S.H., Noh M.Y., Kim S.H., Lee Y.J. (2008). Phosphatidylinositol-3-kinase activation blocks amyloid beta-induced neurotoxicity. Toxicology.

[66] Spires-Jones T.L., Stoothoff W.H., de Calignon A., Jones P.B., Hyman B.T. (2009). Tau pathophysiology in neurodegeneration: A tangled issue. Trends NeuroSci..

[67] Li S.P., Deng Y.Q., Wang X.C., Wang Y.P., Wang J.Z. (2004). Melatonin protects sh-sy5y neuroblastoma cells from calyculin a-induced neurofilament impairment and neurotoxicity. J. Pineal Res..

[68] Yang X., Yang Y., Fu Z., Li Y., Feng J., Luo J., Zhang Q., Wang Q., Tian Q. (2011). Melatonin ameliorates alzheimer-like pathological changes and spatial memory retention impairment induced by calyculin a. J. Psychopharmacol..

[69] Ling Z.Q., Tian Q., Wang L., Fu Z.Q., Wang X.C., Wang Q., Wang J.Z. (2009). Constant illumination induces alzheimer-like damages with endoplasmic reticulum involvement and the protection of melatonin. J. Alzheimers Dis..

[70] Shi C., Zeng J., Li Z., Chen Q., Hang W., Xia L., Wu Y., Chen J., Shi A. (2018). Melatonin mitigates kainic acid-induced neuronal tau hyperphosphorylation and memory deficits through alleviating er stress. Front. Mol. Neurosci..

[71] Wang D.L., Ling Z.Q., Cao F.Y., Zhu L.Q., Wang J.Z. (2004). Melatonin attenuates isoproterenol-induced protein kinase a overactivation and tau hyperphosphorylation in rat brain. J. Pineal Res..

[72] Deng Y.Q., Xu G.G., Duan P., Zhang Q., Wang J.Z. (2005). Effects of melatonin on wortmannin-induced tau hyperphosphorylation. Acta Pharmacol. Sin..

[73] Ferrer I., Gomez-Isla T., Puig B., Freixes M., Ribé E., Dalfó E., Avila J. (2005). Current advances on different kinases involved in tau phosphorylation, and implications in alzheimer’s disease and tauopathies. Curr. Alzheimer Res..

[74] Peng C.X., Hu J., Liu D., Hong X.P., Wu Y.Y., Zhu L.Q., Wang J.Z. (2013). Disease-modified glycogen synthase kinase-3β intervention by melatonin arrests the pathology and memory deficits in an alzheimer’s animal model. Neurobiol. Aging.

[75] Wang S., Zhu L., Shi H., Zheng H., Tian Q., Wang Q., Liu R., Wang J.Z. (2007). Inhibition of melatonin biosynthesis induces neurofilament hyperphosphorylation with activation of cyclin-dependent kinase 5. Neurochem. Res..

[76] Rivera-Bermudez M.A., Gerdin M.J., Earnest D.J., Dubocovich M.L. (2003). Regulation of basal rhythmicity in protein kinase c activity by melatonin in immortalized rat suprachiasmatic nucleus cells. Neurosci. Lett..

[77] Li X.C., Wang Z.F., Zhang J.X., Wang Q., Wang J.Z. (2005). Effect of melatonin on calyculin a-induced tau hyperphosphorylation. Eur. J. Pharmacol..

[78] Zhu L.Q., Wang S.H., Ling Z.Q., Wang D.L., Wang J.Z. (2004). Effect of inhibiting melatonin biosynthesis on spatial memory retention and tau phosphorylation in rat. J. Pineal Res..

[79] Alvira D., Tajes M., Verdaguer E., Acuña-Castroviejo D., Folch J., Camins A., Pallas M. (2006). Inhibition of the cdk5/p25 fragment formation may explain the antiapoptotic effects of melatonin in an experimental model of parkinson’s disease. J. Pineal Res..

[80] Chen D., Mei Y., Kim N., Lan G., Gan C.L., Fan F., Zhang T., Xia Y., Wang L., Lin C. (2020). Melatonin directly binds and inhibits death-associated protein kinase 1 function in alzheimer’s disease. J. Pineal Res..

[81] Kim N., Chen D., Zhou X.Z., Lee T.H. (2019). Death-associated protein kinase 1 phosphorylation in neuronal cell death and neurodegenerative disease. Int. J. Mol. Sci..

[82] Chen D., Zhou X.Z., Lee T.H. (2019). Death-associated protein kinase 1 as a promising drug target in cancer and alzheimer’s disease. Recent Pat. Anti Cancer Drug Discov..

[83] Chen D., Wang L., Lee T.H. (2020). Post-translational modifications of the peptidyl-prolyl isomerase pin1. Front. Cell Dev. Biol..

[84] Wang L., Zhou Y., Chen D., Lee T.H. (2020). Peptidyl-prolyl cis/trans isomerase pin1 and alzheimer’s disease. Front. Cell Dev. Biol..

[85] Jankovic J. (2008). Parkinson’s disease: Clinical features and diagnosis. J. Neurol. Neurosurg. Psychiatry.

[86] Dauer W., Przedborski S. (2003). Parkinson’s disease: Mechanisms and models. Neuron.

[87] Cookson M.R. (2005). The biochemistry of parkinson’s disease. Annu. Rev. Biochem..

[88] Sidhu A., Wersinger C., Moussa C.E., Vernier P. (2004). The role of alpha-synuclein in both neuroprotection and neurodegeneration. Ann. N. Y. Acad. Sci..

[89] Miller D.W., Hague S.M., Clarimon J., Baptista M., Gwinn-Hardy K., Cookson M.R., Singleton A.B. (2004). Alpha-synuclein in blood and brain from familial parkinson disease with snca locus triplication. Neurology.

[90] Spillantini M.G., Crowther R.A., Jakes R., Hasegawa M., Goedert M. (1998). Alpha-synuclein in filamentous inclusions of lewy bodies from parkinson’s disease and dementia with lewy bodies. Proc. Natl. Acad. Sci. USA.

[91] Singhal N.K., Srivastava G., Patel D.K., Jain S.K., Singh M.P. (2011). Melatonin or silymarin reduces maneb- and paraquat-induced parkinson’s disease phenotype in the mouse. J. Pineal Res..

[92] Patki G., Lau Y.S. (2011). Melatonin protects against neurobehavioral and mitochondrial deficits in a chronic mouse model of parkinson’s disease. Pharmacol. Biochem. Behav..

[93] Krüger R., Kuhn W., Müller T., Woitalla D., Graeber M., Kösel S., Przuntek H., Epplen J.T., Schöls L., Riess O. (1998). Ala30pro mutation in the gene encoding alpha-synuclein in parkinson’s disease. Nat. Genet..

[94] Brito-Armas J.M., Baekelandt V., Castro-Hernández J.R., González-Hernández T., Rodríguez M., Castro R. (2013). Melatonin prevents dopaminergic cell loss induced by lentiviral vectors expressing a30p mutant alpha-synuclein. Histol. Histopathol..

[95] Chang C.F., Huang H.J., Lee H.C., Hung K.C., Wu R.T., Lin A.M. (2012). Melatonin attenuates kainic acid-induced neurotoxicity in mouse hippocampus via inhibition of autophagy and α-synuclein aggregation. J. Pineal Res..

[96] Lin A.M., Fang S.F., Chao P.L., Yang C.H. (2007). Melatonin attenuates arsenite-induced apoptosis in rat brain: Involvement of mitochondrial and endoplasmic reticulum pathways and aggregation of alpha-synuclein. J. Pineal Res..

[97] Su L.Y., Li H., Lv L., Feng Y.M., Li G.D., Luo R., Zhou H.J., Lei X.G., Ma L., Li J.L. (2015). Melatonin attenuates mptp-induced neurotoxicity via preventing cdk5-mediated autophagy and snca/α-synuclein aggregation. Autophagy.

[98] Sae-Ung K., Uéda K., Govitrapong P., Phansuwan-Pujito P. (2012). Melatonin reduces the expression of alpha-synuclein in the dopamine containing neuronal regions of amphetamine-treated postnatal rats. J. Pineal Res..

[99] Zampol M.A., Barros M.H. (2018). Melatonin improves survival and respiratory activity of yeast cells challenged by alpha-synuclein and menadione. Yeast.

[100] Adi N., Mash D.C., Ali Y., Singer C., Shehadeh L., Papapetropoulos S. (2010). Melatonin mt1 and mt2 receptor expression in parkinson’s disease. Med. Sci. Monit..

[101] Willis G.L. (2008). Parkinson’s disease as a neuroendocrine disorder of circadian function: Dopamine-melatonin imbalance and the visual system in the genesis and progression of the degenerative process. Rev. Neurosci..

[102] Alexiuk N.A., Vriend J.P. (1993). Melatonin reduces dopamine content in the neurointermediate lobe of male syrian hamsters. Brain Res. Bull..

[103] Willis G.L. (2005). The role of ml-23 and other melatonin analogues in the treatment and management of parkinson’s disease. Drug News Perspect..

[104] Willis G.L. (2008). Intraocular microinjections repair experimental parkinson’s disease. Brain Res..

[105] Ross C.A., Tabrizi S.J. (2011). Huntington’s disease: From molecular pathogenesis to clinical treatment. Lancet Neurol..

[106] van der Burg J.M., Björkqvist M., Brundin P. (2009). Beyond the brain: Widespread pathology in huntington’s disease. Lancet Neurol..

[107] Sawa A., Wiegand G.W., Cooper J., Margolis R.L., Sharp A.H., Lawler J.F.J., Greenamyre J.T., Snyder S.H., Ross C.A. (1999). Increased apoptosis of huntington disease lymphoblasts associated with repeat length-dependent mitochondrial depolarization. Nat. Med..

[108] Kalliolia E., Silajdžić E., Nambron R., Hill N.R., Doshi A., Frost C., Watt H., Hindmarsh P., Björkqvist M., Warner T.T. (2014). Plasma melatonin is reduced in huntington’s disease. Mov. Disord..

[109] Xue F., Shi C., Chen Q., Hang W., Xia L., Wu Y., Tao S.Z., Zhou J., Shi A., Chen J. (2017). Melatonin mediates protective effects against kainic acid-induced neuronal death through safeguarding er stress and mitochondrial disturbance. Front. Mol. Neurosci..

[110] Tan D.X., Manchester L.C., Reiter R.J., Qi W., Kim S.J., El-Sokkary G.H. (1998). Melatonin protects hippocampal neurons in vivo against kainic acid-induced damage in mice. J. Neurosci. Res..

[111] Manev H., Uz T., Kharlamov A., Cagnoli C.M., Franceschini D., Giusti P. (1996). In vivo protection against kainate-induced apoptosis by the pineal hormone melatonin: Effect of exogenous melatonin and circadian rhythm. Restor. Neurol. Neurosci..

[112] Túnez I., Montilla P., Del Carmen Muñoz M., Feijóo M., Salcedo M. (2004). Protective effect of melatonin on 3-nitropropionic acid-induced oxidative stress in synaptosomes in an animal model of huntington’s disease. J. Pineal Res..

[113] Nam E., Lee S.M., Koh S.E., Joo W.S., Maeng S., Im H.I., Kim Y.S. (2005). Melatonin protects against neuronal damage induced by 3-nitropropionic acid in rat striatum. Brain Res..

[114] Mu S., Lin E., Liu B., Ma Y., OuYang L., Li Y., Chen S., Zhang J., Lei W. (2014). Melatonin reduces projection neuronal injury induced by 3-nitropropionic acid in the rat striatum. Neuro Degener. Dis..

[115] Mochel F., Haller R.G. (2011). Energy deficit in huntington disease: Why it matters. J. Clin. Investig..

[116] Wang X., Sirianni A., Pei Z., Cormier K., Smith K., Jiang J., Zhou S., Wang H., Zhao R., Yano H. (2011). The melatonin mt1 receptor axis modulates mutant huntingtin-mediated toxicity. J. Neurosci..

[117] Zhang L., Li F., Su X., Li Y., Wang Y., Fang R., Guo Y., Jin T., Shan H., Zhao X. (2019). Melatonin prevents lung injury by regulating apelin 13 to improve mitochondrial dysfunction. Exp. Mol. Med..

[118] Ruiz A., Matute C., Alberdi E. (2010). Intracellular ca^2+^ release through ryanodine receptors contributes to ampa receptor-mediated mitochondrial dysfunction and er stress in oligodendrocytes. Cell Death Dis..

[119] Bano D., Zanetti F., Mende Y., Nicotera P. (2011). Neurodegenerative processes in huntington’s disease. Cell Death Dis..

[120] Leung A.W., Halestrap A.P. (2008). Recent progress in elucidating the molecular mechanism of the mitochondrial permeability transition pore. Biochim. Biophys. Acta.

[121] Andrabi S.A., Sayeed I., Siemen D., Wolf G., Horn T.F. (2004). Direct inhibition of the mitochondrial permeability transition pore: A possible mechanism responsible for anti-apoptotic effects of melatonin. FASEB J. Off. Publ. Fed. Am. Soc. Exp. Biol..

[122] Wang X. (2009). The antiapoptotic activity of melatonin in neurodegenerative diseases. CNS Neurosci. Ther..

[123] Mohseni M., Mihandoost E., Shirazi A., Sepehrizadeh Z., Bazzaz J.T., Ghazi-khansari M. (2012). Melatonin may play a role in modulation of bax and bcl-2 expression levels to protect rat peripheral blood lymphocytes from gamma irradiation-induced apoptosis. Mutat. Res..

[124] Radogna F., Cristofanon S., Paternoster L., D’Alessio M., De Nicola M., Cerella C., Dicato M., Diederich M., Ghibelli L. (2008). Melatonin antagonizes the intrinsic pathway of apoptosis via mitochondrial targeting of bcl-2. J. Pineal Res..

[125] Radogna F., Albertini M.C., De Nicola M., Diederich M., Bejarano I., Ghibelli L. (2015). Melatonin promotes bax sequestration to mitochondria reducing cell susceptibility to apoptosis via the lipoxygenase metabolite 5-hydroxyeicosatetraenoic acid. Mitochondrion.

[126] Ghasemi N., Razavi S., Nikzad E. (2017). Multiple sclerosis: Pathogenesis, symptoms, diagnoses and cell-based therapy. Cell J..

[127] Kamm C.P., Uitdehaag B.M., Polman C.H. (2014). Multiple sclerosis: Current knowledge and future outlook. Eur. Neurol..

[128] Wootla B., Eriguchi M., Rodriguez M. (2012). Is multiple sclerosis an autoimmune disease?. Autoimmune Dis..

[129] Compston A., Coles A. (2008). Multiple sclerosis. Lancet.

[130] Akpinar Z., Tokgöz S., Gökbel H., Okudan N., Uğuz F., Yilmaz G. (2008). The association of nocturnal serum melatonin levels with major depression in patients with acute multiple sclerosis. Psychiatry Res..

[131] Farhadi N., Oryan S., Nabiuni M. (2014). Serum levels of melatonin and cytokines in multiple sclerosis. Biomed. J..

[132] Damasceno A., Moraes A.S., Farias A., Damasceno B.P., dos Santos L.M., Cendes F. (2015). Disruption of melatonin circadian rhythm production is related to multiple sclerosis severity: A preliminary study. J. Neurol. Sci..

[133] Melamud L., Golan D., Luboshitzky R., Lavi I., Miller A. (2012). Melatonin dysregulation, sleep disturbances and fatigue in multiple sclerosis. J. Neurol. Sci..

[134] Abo Taleb H.A., Alghamdi B.S. (2020). Neuroprotective effects of melatonin during demyelination and remyelination stages in a mouse model of multiple sclerosis. J. Mol. Neurosci. MN.

[135] Vakilzadeh G., Khodagholi F., Ghadiri T., Ghaemi A., Noorbakhsh F., Sharifzadeh M., Gorji A. (2016). The effect of melatonin on behavioral, molecular, and histopathological changes in cuprizone model of demyelination. Mol. Neurobiol..

[136] Kashani I.R., Rajabi Z., Akbari M., Hassanzadeh G., Mohseni A., Eramsadati M.K., Rafiee K., Beyer C., Kipp M., Zendedel A. (2014). Protective effects of melatonin against mitochondrial injury in a mouse model of multiple sclerosis. Exp. Brain Res..

[137] Mascanfroni I.D., Yeste A., Vieira S.M., Burns E.J., Patel B., Sloma I., Wu Y., Mayo L., Ben-Hamo R., Efroni S. (2013). Il-27 acts on dcs to suppress the t cell response and autoimmunity by inducing expression of the immunoregulatory molecule cd39. Nat. Immunol..

[138] Álvarez-Sánchez N., Cruz-Chamorro I., Díaz-Sánchez M., Sarmiento-Soto H., Medrano-Campillo P., Martínez-López A., Lardone P.J., Guerrero J.M., Carrillo-Vico A. (2017). Melatonin reduces inflammatory response in peripheral t helper lymphocytes from relapsing-remitting multiple sclerosis patients. J. Pineal Res..

[139] Farez M.F., Mascanfroni I.D., Méndez-Huergo S.P., Yeste A., Murugaiyan G., Garo L.P., Balbuena Aguirre M.E., Patel B., Ysrraelit M.C., Zhu C. (2015). Melatonin contributes to the seasonality of multiple sclerosis relapses. Cell.

[140] von Lewinski F., Keller B.U. (2005). Ca^2+^, mitochondria and selective motoneuron vulnerability: Implications for als. Trends Neurosci..

[141] Zhang Y., Cook A., Kim J., Baranov S.V., Jiang J., Smith K., Cormier K., Bennett E., Browser R.P., Day A.L. (2013). Melatonin inhibits the caspase-1/cytochrome c/caspase-3 cell death pathway, inhibits mt1 receptor loss and delays disease progression in a mouse model of amyotrophic lateral sclerosis. Neurobiol. Dis..

[142] Rogério F., Teixeira S.A., de Rezende A.C., de Sá R.C., de Souza Queiroz L., De Nucci G., Muscará M.N., Langone F. (2005). Superoxide dismutase isoforms 1 and 2 in lumbar spinal cord of neonatal rats after sciatic nerve transection and melatonin treatment. Brain Res. Dev. Brain Res..

[143] Hirano A., Donnenfeld H., Sasaki S., Nakano I. (1984). Fine structural observations of neurofilamentous changes in amyotrophic lateral sclerosis. J. Neuropathol. Exp. Neurol..

[144] Rouleau G.A., Clark A.W., Rooke K., Pramatarova A., Krizus A., Suchowersky O., Julien J.P., Figlewicz D. (1996). Sod1 mutation is associated with accumulation of neurofilaments in amyotrophic lateral sclerosis. Ann. Neurol..

[145] Julien J.P., Beaulieu J.M. (2000). Cytoskeletal abnormalities in amyotrophic lateral sclerosis: Beneficial or detrimental effects?. J. Neurol. Sci..

[146] Estévez A.G., Crow J.P., Sampson J.B., Reiter C., Zhuang Y., Richardson G.J., Tarpey M.M., Barbeito L., Beckman J.S. (1999). Induction of nitric oxide-dependent apoptosis in motor neurons by zinc-deficient superoxide dismutase. Science.

[147] Crow J.P., Sampson J.B., Zhuang Y., Thompson J.A., Beckman J.S. (1997). Decreased zinc affinity of amyotrophic lateral sclerosis-associated superoxide dismutase mutants leads to enhanced catalysis of tyrosine nitration by peroxynitrite. J. Neurochem..

[148] Schiavon A.P., Soares L.M., Bonato J.M., Milani H., Guimarães F.S., Weffort de Oliveira R.M. (2014). Protective effects of cannabidiol against hippocampal cell death and cognitive impairment induced by bilateral common carotid artery occlusion in mice. Neurotox. Res..

[149] Meyer J.S., Rauch G., Rauch R.A., Haque A. (2000). Risk factors for cerebral hypoperfusion, mild cognitive impairment, and dementia. Neurobiol. Aging.

[150] Kalaria R.N., Maestre G.E., Arizaga R., Friedland R.P., Galasko D., Hall K., Luchsinger J.A., Ogunniyi A., Perry E.K., Potocnik F. (2008). Alzheimer’s disease and vascular dementia in developing countries: Prevalence, management, and risk factors. Lancet Neurol..

[151] Wimo A., Guerchet M., Ali G.C., Wu Y.T., Prina A.M., Winblad B., Jönsson L., Liu Z., Prince M. (2017). The worldwide costs of dementia 2015 and comparisons with 2010. Alzheimer’s Dement..

[152] Xi Y., Wang M., Zhang W., Bai M., Du Y., Zhang Z., Li Z., Miao J. (2014). Neuronal damage, central cholinergic dysfunction and oxidative damage correlate with cognitive deficits in rats with chronic cerebral hypoperfusion. Neurobiol. Learn. Mem..

[153] Gupta S., Singh P., Sharma B.M., Sharma B. (2015). Neuroprotective effects of agomelatine and vinpocetine against chronic cerebral hypoperfusion induced vascular dementia. Curr. Neurovascular Res..

[154] Bin-Jaliah I., Sakr H.F. (2018). Melatonin ameliorates brain oxidative stress and upregulates senescence marker protein-30 and osteopontin in a rat model of vascular dementia. Physiol. Int..

[155] Cardinali D.P., Nagle C.A., Freire F., Rosner J.M. (1975). Effects of melatonin on neurotransmitter uptake and release by synaptosome-rich homogenates of the rat hypothalamus. Neuroendocrinology.

[156] Niizuma K., Endo H., Chan P.H. (2009). Oxidative stress and mitochondrial dysfunction as determinants of ischemic neuronal death and survival. J. Neurochem..

[157] Das A., McDowell M., Pava M.J., Smith J.A., Reiter R.J., Woodward J.J., Varma A.K., Ray S.K., Banik N.L. (2010). The inhibition of apoptosis by melatonin in vsc4.1 motoneurons exposed to oxidative stress, glutamate excitotoxicity, or tnf-alpha toxicity involves membrane melatonin receptors. J. Pineal Res..

[158] Ali T., Badshah H., Kim T.H., Kim M.O. (2015). Melatonin attenuates d-galactose-induced memory impairment, neuroinflammation and neurodegeneration via rage/nf-k b/jnk signaling pathway in aging mouse model. J. Pineal Res..

[159] Mauriz J.L., Collado P.S., Veneroso C., Reiter R.J., González-Gallego J. (2013). A review of the molecular aspects of melatonin’s anti-inflammatory actions: Recent insights and new perspectives. J. Pineal Res..

[160] Ishigami A., Handa S., Maruyama N., Supakar P.C. (2003). Nuclear localization of senescence marker protein-30, smp30, in cultured mouse hepatocytes and its similarity to rna polymerase. Biosci. Biotechnol. Biochem..

[161] Yun S.P., Han Y.S., Lee J.H., Kim S.M., Lee S.H. (2018). Melatonin rescues mesenchymal stem cells from senescence induced by the uremic toxin p-cresol via inhibiting mtor-dependent autophagy. Biomol. Ther..

[162] Meller R., Stevens S.L., Minami M., Cameron J.A., King S., Rosenzweig H., Doyle K., Lessov N.S., Simon R.P., Stenzel-Poore M.P. (2005). Neuroprotection by osteopontin in stroke. J. Cereb. Blood Flow Metab..

[163] Suzuki H., Ayer R., Sugawara T., Chen W., Sozen T., Hasegawa Y., Kanamaru K., Zhang J.H. (2010). Protective effects of recombinant osteopontin on early brain injury after subarachnoid hemorrhage in rats. Crit. Care Med..

[164] Chen W., Ma Q., Suzuki H., Hartman R., Tang J., Zhang J.H. (2011). Osteopontin reduced hypoxia-ischemia neonatal brain injury by suppression of apoptosis in a rat pup model. Stroke.

[165] Yang Y., Jiang S., Dong Y., Fan C., Zhao L., Yang X., Li J., Di S., Yue L., Liang G. (2015). Melatonin prevents cell death and mitochondrial dysfunction via a sirt1-dependent mechanism during ischemic-stroke in mice. J. Pineal Res..

[166] Feng Z., Cheng Y., Zhang J.T. (2004). Long-term effects of melatonin or 17 beta-estradiol on improving spatial memory performance in cognitively impaired, ovariectomized adult rats. J. Pineal Res..

[167] Shen D., Tian X., Sang W., Song R. (2016). Effect of melatonin and resveratrol against memory impairment and hippocampal damage in a rat model of vascular dementia. Neuroimmunomodulation.

[168] Leal G., Comprido D., Duarte C.B. (2014). Bdnf-induced local protein synthesis and synaptic plasticity. Neuropharmacology.

[169] Ninan I. (2014). Synaptic regulation of affective behaviors; role of bdnf. Neuropharmacology.

[170] Imbesi M., Uz T., Dzitoyeva S., Manev H. (2008). Stimulatory effects of a melatonin receptor agonist, ramelteon, on bdnf in mouse cerebellar granule cells. Neurosci. Lett..

[171] Brusco L.I., Márquez M., Cardinali D.P. (1998). Monozygotic twins with alzheimer’s disease treated with melatonin: Case report. J. Pineal Res..

[172] Khachiyants N., Trinkle D., Son S.J., Kim K.Y. (2011). Sundown syndrome in persons with dementia: An update. Psychiatry Investig..

[173] Klaffke S., Staedt J. (2006). Sundowning and circadian rhythm disorders in dementia. Acta Neurol. Belg..

[174] Lammers M., Ahmed A.I. (2013). Melatonin for sundown syndrome and delirium in dementia: Is it effective?. J. Am. Geriatrics Soc..

[175] Anderson K.N., Jamieson S., Graham A.J., Shneerson J.M. (2008). Rem sleep behaviour disorder treated with melatonin in a patient with alzheimer’s disease. Clin. Neurol. Neurosurg..

[176] Jean-Louis G., Zizi F., von Gizycki H., Taub H. (1998). Effects of melatonin in two individuals with alzheimer’s disease. Percept. Mot. Ski..

[177] Wade A.G., Farmer M., Harari G., Fund N., Laudon M., Nir T., Frydman-Marom A., Zisapel N. (2014). Add-on prolonged-release melatonin for cognitive function and sleep in mild to moderate alzheimer’s disease: A 6-month, randomized, placebo-controlled, multicenter trial. Clin. Interv. Aging.

[178] Alves G.S., Carvalho A.F., de Amorim de Carvalho L., Sudo F.K., Siqueira-Neto J.I., Oertel-Knochel V., Jurcoane A., Knochel C., Boecker H., Laks J. (2017). Neuroimaging findings related to behavioral disturbances in alzheimer’s disease: A systematic review. Curr. Alzheimer Res..

[179] Serfaty M., Kennell-Webb S., Warner J., Blizard R., Raven P. (2002). Double blind randomised placebo controlled trial of low dose melatonin for sleep disorders in dementia. Int. J. Geriatr. Psychiatry.

[180] Singer C., Tractenberg R.E., Kaye J., Schafer K., Gamst A., Grundman M., Thomas R., Thal L.J. (2003). A multicenter, placebo-controlled trial of melatonin for sleep disturbance in alzheimer’s disease. Sleep.

[181] Asayama K., Yamadera H., Ito T., Suzuki H., Kudo Y., Endo S. (2003). Double blind study of melatonin effects on the sleep-wake rhythm, cognitive and non-cognitive functions in alzheimer type dementia. J. Nippon Med. Sch..

[182] Fainstein I., Bonetto A.J., Brusco L.I., Cardinali D.P. (1997). Effects of melatonin in elderly patients with sleep disturbance: A pilot study. Curr. Ther. Res..

[183] Cohen-Mansfield J., Garfinkel D., Lipson S. (2000). Melatonin for treatment of sundowning in elderly persons with dementia—A preliminary study. Arch. Gerontol Geriatr..

[184] Mishima K., Okawa M., Hozumi S., Hishikawa Y. (2000). Supplementary administration of artificial bright light and melatonin as potent treatment for disorganized circadian rest-activity and dysfunctional autonomic and neuroendocrine systems in institutionalized demented elderly persons. Chronobiol. Int..

[185] Cardinali D.P., Brusco L.I., Liberczuk C., Furio A.M. (2002). The use of melatonin in alzheimer’s disease. Neuro Endocrinol. Lett..

[186] Mahlberg R., Walther S. (2007). Actigraphy in agitated patients with dementia. Monitoring treatment outcomes. Z. Gerontol. Geriatr..

[187] Cardinali D.P., Furio A.M., Brusco L.I. (2010). Clinical aspects of melatonin intervention in alzheimer’s disease progression. Curr. Neuropharmacol..

[188] Cardinali D.P., Vigo D.E., Olivar N., Vidal M.F., Brusco L.I. (2014). Melatonin therapy in patients with alzheimer’s disease. Antioxidants.

[189] de Jonghe A., Korevaar J.C., van Munster B.C., de Rooij S.E. (2010). Effectiveness of melatonin treatment on circadian rhythm disturbances in dementia. Are there implications for delirium? A systematic review. Int. J. Geriatr. Psychiatry.

[190] Sanchez-Barcelo E.J., Rueda N., Mediavilla M.D., Martinez-Cue C., Reiter R.J. (2017). Clinical uses of melatonin in neurological diseases and mental and behavioural disorders. Curr. Med. Chem..

[191] Pandi-Perumal S.R., BaHammam A.S., Brown G.M., Spence D.W., Bharti V.K., Kaur C., Hardeland R., Cardinali D.P. (2013). Melatonin antioxidative defense: Therapeutical implications for aging and neurodegenerative processes. Neurotox. Res..

[192] Dowling G.A., Burr R.L., Van Someren E.J., Hubbard E.M., Luxenberg J.S., Mastick J., Cooper B.A. (2008). Melatonin and bright-light treatment for rest-activity disruption in institutionalized patients with alzheimer’s disease. J. Am. Geriatr. Soc..

[193] Riemersma-van der Lek R.F., Swaab D.F., Twisk J., Hol E.M., Hoogendijk W.J., Van Someren E.J. (2008). Effect of bright light and melatonin on cognitive and noncognitive function in elderly residents of group care facilities: A randomized controlled trial. JAMA.

[194] Bordet R., Devos D., Brique S., Touitou Y., Guieu J.D., Libersa C., Destée A. (2003). Study of circadian melatonin secretion pattern at different stages of parkinson’s disease. Clin. Neuropharmacol..

[195] Dowling G.A., Mastick J., Colling E., Carter J.H., Singer C.M., Aminoff M.J. (2005). Melatonin for sleep disturbances in parkinson’s disease. Sleep Med..

[196] Medeiros C.A., Carvalhedo de Bruin P.F., Lopes L.A., Magalhães M.C., de Lourdes Seabra M., de Bruin V.M. (2007). Effect of exogenous melatonin on sleep and motor dysfunction in parkinson’s disease. A randomized, double blind, placebo-controlled study. J. Neurol..

[197] Dowling G., Mastick J., Aminoff M. (2003). Melatonin for sleep disturbances in parkinson’s disease: A pilot study. Sleep Res. Online.

[198] Litvinenko I.V., Krasakov I.V., Tikhomirova O.V. (2012). [sleep disorders in parkinson’s disease without dementia: A comparative randomized controlled study of melatonin and clonazepam]. Zhurnal Nevrologii Psikhiatrii Imeni S.S. Korsakova.

[199] Datieva V.K., Rosinskaia A.V., Levin O.S. (2013). [the use of melatonin in the treatment of chronic fatigue syndrome and circadian rhythm disorders in parkinson’s disease]. Zhurnal Nevrologii Psikhiatrii Imeni S.S. Korsakova.

[200] Jacob S., Poeggeler B., Weishaupt J.H., Sirén A.L., Hardeland R., Bähr M., Ehrenreich H. (2002). Melatonin as a candidate compound for neuroprotection in amyotrophic lateral sclerosis (als): High tolerability of daily oral melatonin administration in als patients. J. Pineal Res..

[201] Adamczyk-Sowa M., Pierzchala K., Sowa P., Polaniak R., Kukla M., Hartel M. (2014). Influence of melatonin supplementation on serum antioxidative properties and impact of the quality of life in multiple sclerosis patients. J. Physiol. Pharmacol..

[202] Skarlis C., Anagnostouli M. (2020). The role of melatonin in multiple sclerosis. Neurol. Sci..

[203] Hardeland R. (2012). Melatonin in aging and disease -multiple consequences of reduced secretion, options and limits of treatment. Aging Dis..

[204] Turek F.W., Gillette M.U. (2004). Melatonin, sleep, and circadian rhythms: Rationale for development of specific melatonin agonists. Sleep Med..

[205] Hardeland R. (2010). Investigational melatonin receptor agonists. Expert Opin. Investig. Drugs.

[206] Spadoni G., Bedini A., Rivara S., Mor M. (2011). Melatonin receptor agonists: New options for insomnia and depression treatment. CNS Neurosci. Ther..

